# Understanding citizens’ preferences for prioritising patients in the face of scarce surgical capacity in the Netherlands: a think-aloud study

**DOI:** 10.1136/bmjopen-2025-115112

**Published:** 2026-07-07

**Authors:** Thomas Reindersma, Anouk van Alphen, Vivian Reckers-Droog

**Affiliations:** 1 Department of Health Services Management & Organisation, Erasmus Universiteit Rotterdam, Erasmus School of Health Policy and Management, Rotterdam, The Netherlands; 2 Department of Otorhinolaryngology, Erasmus MC, Rotterdam, The Netherlands; 3 Department of Health Economics, Erasmus Universiteit Rotterdam, Erasmus School of Health Policy and Management, Rotterdam, The Netherlands

**Keywords:** Decision Making, Health economics, Health policy, Rationing

## Abstract

**Abstract:**

**Objectives:**

Scarce surgical capacity makes prioritisation decisions unavoidable. During crises, the complexity and societal sensitivity of such decisions become particularly evident, underscoring the importance of engaging multiple stakeholders, including citizens. Citizens may hold different preferences and attach importance to different considerations than physicians regarding what is fair or appropriate when allocating scarce surgical capacity. This study, therefore, examined which preferences and considerations citizens consider important in decisions about prioritising patients for limited surgical capacity.

**Design:**

We conducted semi-structured think-aloud interviews in which participants completed two sets of 12 labelled choice tasks derived from a discrete choice experiment. In the first set, they prioritised one of three patients with different conditions (breast cancer, deafness and knee arthrosis) for limited surgical capacity. In the second, they prioritised one of two patients with the same condition (breast cancer, deafness or knee arthrosis).

**Setting:**

The setting of the study was in the Netherlands.

**Participants:**

25 citizens were purposively sampled.

**Results:**

We developed a framework comprising 13 categories grouped under five themes that provided insight into how participants (A) performed the choice tasks, (B) perceived the patients, (C) perceived the conditions, (D) perceived the treatment options and (E) stated a preference.

**Conclusions:**

Our study shows that rather than relying solely on the attributes presented in the choice tasks, participants integrated a wide array of contextual, emotional and ethical factors into their decision-making. Perceived disease severity was of specific importance to citizens. Our findings further suggest that citizens not only draw from principles of utilitarianism but also draw from principles of prioritarianism.

**STRENGTHS AND LIMITATIONS OF THIS STUDY**

- The combined use of discrete choice experiment-based choice tasks and think-aloud methodology enabled insight into how citizens reasoned about prioritising patients for scarce surgical care.
- Inclusion of diverse medical conditions (breast cancer, deafness and knee arthrosis) ensured variation in patient, disease and contextual characteristics, strengthening the study’s ability to capture a broad range of preferences and prioritisation considerations.
- An overrepresentation of tertiary-educated participants, underrepresentation of adults aged 65–80 and no inclusion of individuals with lower education levels may restrict the generalisability of the findings.

## Introduction

 The demand for healthcare continues to rise because of ageing populations, increased longevity and the growing prevalence of chronic conditions. This puts increasing pressure on available healthcare resources, which becomes particularly apparent during epidemics, pandemics and other crises—when already limited resources are under severe strain—as demonstrated by the COVID-19 pandemic.

During this pandemic, surgical capacity in hospitals in the Netherlands was significantly limited due to shortages of medical staff and intensive care unit (ICU) beds needed for post-operative care. In this country alone, over 300 000 surgeries were postponed in 2020 and 2021, leading to longer waiting times and negative impacts on patient health.^1 2^ As a result, prioritisation of patients for surgical care became unavoidable.^2^

A decision model was developed to provide physicians with objective and transparent support in making prioritisation decisions.^3^ The model quantifies the health impact of surgical delay and the substitution of surgery with alternative non-surgical treatments. By using this model outcome as an urgency measure, health losses can be minimised at the societal level.^3^ Recently, the decision model was extended to quantify the health impact of substituting surgery with non-surgical alternatives for 11 conditions where such alternatives are available (see online supplemental material S1 for an overview).^4^ While such a decision model may aid physicians in making prioritisation decisions, prioritisation involves more than just applying a decision model.^5^ Indeed, it is a complex process that requires balancing medical, ethical and societal considerations, as well as engaging multiple stakeholders, including citizens.^6 7^

Citizens may have different preferences and deem other considerations important than do physicians about what is fair or appropriate when allocating scarce surgical capacity. This became apparent in the Netherlands during the COVID-19 pandemic, when the Federation of Medical Specialists and the Royal Dutch Society for the Advancement of Medicine proposed the ICU prioritisation guideline ‘Triage based on non-medical considerations for ICU admission in phase III of the COVID-19 pandemic’ (colloquially referred to as Code Black).^8^ News about this guideline in the media resulted in public outcry and sparked a public debate,^9^ highlighting that prioritisation decisions during times of extreme scarcity are particularly sensitive to public opinion.

Given the possibility of future crises—such as pandemics—that may lead to significant scarcity in surgical capacity, gaining deeper insight into citizens’ views and preferences is important. The aim of this study was therefore to examine citizens’ preferences and the considerations they regard as important in decisions on the prioritisation of patients for scarce surgical capacity. While quantitative preference elicitation methods such as discrete choice experiments (DCEs) are well suited to estimate the relative importance of attributes and preference weights, a qualitative think-aloud approach was considered most appropriate for the present study because it enables in-depth exploration of how participants interpret information, make trade-offs and reason about prioritisation decisions. To meet this aim, we conducted a qualitative think-aloud study among adult citizens in the Netherlands, in which participants completed choice tasks derived from a DCE while ‘thinking aloud’. In a qualitative think-aloud study, participants are asked to verbalise their thoughts while, for example, in the current study, completing choice tasks to gain insight into how they interpret and weigh different attributes, make trade-offs and reflect on what matters to them in the context of scarce surgical capacity.^10^,^12^ The choice tasks focused on prioritising patients suffering from breast cancer, deafness and knee arthrosis. These three conditions were selected from a set of 11 conditions included in the aforementioned decision model^4^ due to markedly different characteristics across patients, conditions and contextual factors. This allowed us to gain insight into a broad range of preferences and considerations. The findings of this study may be of interest to those aiming to align prioritisation decisions more closely with citizens’ preferences and considerations. In addition, the findings may be of interest to researchers aiming to use think-aloud methods to complement the development and interpretation of DCEs by gaining insight into how participants interpret attributes, weigh considerations and reason through prioritisation decisions.

## Materials and methods

### Sample and data collection

We conducted the think-aloud study with 25 citizens in the Netherlands in June 2023. The participants were recruited by Motivaction, an independent research agency, and purposively sampled based on age (18+ years), sex, educational level and whether they had children (of any age). We anticipated that these characteristics could influence the participants’ preferences for prioritising patients for scarce surgical care, based on the findings of previous studies focusing on prioritisation in healthcare.^13^,^16^ To further increase the likelihood of capturing a broad range of considerations across the sample, we stratified based on participants’ Mentality profile.^17^ These (eight) profiles, developed by Motivaction, each represent a distinct set of shared values regarding work, leisure and politics, as well as distinct lifestyle and consumption patterns (see online supplemental material S2 for a brief description of each profile and the distribution of profiles across participants).

The interviews were all conducted in person in the same location (ie, a university campus) by the first author (TR), a male PhD candidate with an MSc degree and prior training and experience in conducting qualitative interviews. No prior relationship existed between the interviewer and participants, and no individuals other than the interviewer and participant were present during the interviews. Based on the sample size of previous think-aloud studies,^18 19^ we anticipated that 25 interviews would suffice to reach data saturation. Prior to the interviews, we informed participants that surgical capacity in Dutch hospitals is limited. Capacity has been under pressure since the COVID-19 pandemic and is expected to remain so mainly due to staff shortages and the growing needs of an ageing population. We explained that physicians must, therefore, decide which patients can undergo surgery and which must wait. In doing so, participants consider factors such as the patient’s age, health status before and after surgery and time spent on the waiting list. We also explained the aim of the study—to gain insight into the prioritisation preferences and the underlying considerations of members of the public in the Netherlands—as well as the interview protocol.

Before the interviews took place, we explained the reasons for conducting the study and obtained written consent from all participants to audio-record the interviews and use their anonymised data for the purpose of this study. Participants received a fee of 55 euros to compensate for their time and travel expenses. Ethical approval was obtained from the internal review board of Erasmus School of Health Policy & Management (reference: ETH2223-0498).

#### Public involvement

The sampled citizens participated in this study as interview participants. However, they were not involved in the design, conduct, reporting or dissemination plans of the research.

### Interview protocol

The interviews were conducted using a standardised interview protocol that can be found in online supplemental material S3. The participants performed the choice tasks on a laptop, while the interviewer sat diagonally across from the participant and observed the participant’s activities on a second screen suspended above the table. During the interview, participants were asked to verbalise their thoughts while completing two sets of 12 choice tasks and were reminded of this when remaining silent.^12 18^

Before completing the choice tasks, we asked participants to indicate their own health-related quality of life (HRQoL) using the EQ-5D-5L and EQ-5D visual analogue scale (VAS) ranging from 0 (‘Worst health you can imagine’) to 100 (‘Best health you can imagine’) (EuroQol Research Foundation registration number: 84521). We then introduced participants, in three steps, to the conditions (ie, breast cancer, deafness and knee arthrosis), the surgical and alternative treatment options and the attributes and levels used in the choice tasks: age, HRQoL before surgery (measured in points on the EQ-5D VAS), HRQoL after surgery (measured in points on the EQ-5D VAS) and waiting time until surgery. Table 1 presents an overview of the conditions, attributes and levels.

**Table 1:** Overview of conditions, attributes and levels

Attribute	Condition		
	Breast cancer	Deafness	Knee arthrosis
Age (in years)*	50; 60; 70	0.5; 0.75; 1.0	60; 70; 80
HRQoL before surgery (in points on EQ-5D VAS)	65; 75; 85	65; 75; 85	65; 75; 85
HRQoL after surgery (in points on EQ-5D VAS)	80; 85; 90	80; 85; 90	80; 85; 90
Waiting time (in months)	1; 3; 6	1; 3; 6	1; 3; 6

* Note that age was presented as 6, 9 or 12 months for patients suffering from deafness.

HRQoL, health-related quality of life; VAS, visual analogue scale.

After each step, we asked participants to complete a practice choice task, which gradually increased in complexity, leading up to the first set of 12 choice tasks. After participants completed each set of choice tasks while thinking aloud, we asked them to reflect on their choices and share any thoughts they had not yet articulated but considered important to mention.^20^

### Choice tasks derived from a discrete choice experiment

The choice tasks were used as an elicitation tool to explore how participants reasoned about prioritisation decisions and interpreted the attributes included in the tasks. The DCE was split into two sets of 12 choice tasks, without the option to opt out of individual tasks. The first set of tasks consisted of choosing which out of three patients with a different condition (ie, breast cancer, deafness or knee arthrosis) should be prioritised for surgical care (see table 2 for an example choice task). The second set of tasks consisted of choosing which out of two patients with the same condition (ie, either breast cancer, deafness or knee arthrosis) should be prioritised for surgical care. This condition was randomly selected.

**Table 2:** Example choice task (set 1, block 1, choice task 6)

	Patient 1 suffers from breast cancer	Patient 2 suffers from deafness	Patient 3 suffers from knee arthrosis
Age	70 years	6 months	70 years
Quality of life before surgery (0–100 scale)	85 points	75 points	65 points
Quality of life during 1 year after surgery (0–100 scale)	90 points	80 points	80 points
Waiting time	1 month	3 months	18 months
Which patient should be prioritised for surgery?	q	q	q

We used informed priors and Ngene V.1.4^21^ to construct four Bayesian D-efficient designs (ie, one ‘between-conditions’ design and three ‘within- condition’ designs), each consisting of 48 choice tasks. The designs were divided into four blocks while retaining level balance so that each block consisted of 12 choice tasks. The blocks and the order of the choice tasks were assigned randomly to patients. The experimental designs are included as online supplemental material S4.

Note that the findings of this qualitative think-aloud study informed the refinement and interpretation of a subsequent large-scale DCE study, the results of which have been published elsewhere.^22^

### Data analysis

To analyse the interview data, a framework analysis approach was employed.^23^ Framework analysis enabled us to uncover themes in the qualitative data, such as how participants verbally expressed their decision-making processes and the mental steps they take to reach a conclusion and state a preference. This approach leaves open the possibility of unanticipated insights. The framework analysis consisted of transcription, familiarisation with the data, inductive coding, developing an analytical framework, application of the framework to the data set and finally charting and interpreting the data.^23^ Data were coded by the first author in Atlas.ti 24.0. The second author coded a random selection of 10 transcripts to refine the analytical framework and enhance coding reliability. This was done in two iterations. First, the second author coded five transcripts. After discussion with the first author, another five transcripts were coded.

## Results

Online supplemental material S5.1 presents the sample characteristics. On average, the participants were 46 years old. Most participants were female (n=13), had a high education level (n=16), did not have children (n=13) and were employed (n=19). On average, their HRQoL was 0.92 and 83.4, as measured on the EQ-5D-5L and EQ-5D VAS, respectively. The interviews lasted on average 53 min, and data saturation was reached after 20 interviews.

Based on the framework analysis, we identified 13 categories grouped under the five themes: (A) performing the choice tasks, (B) perceptions about the patients, (C) perceptions about the condition, (D) perceptions about the treatment options and (E) stating a preference. Table 3 presents an overview of the themes and corresponding categories. A narrative summary with illustrative quotes from participants (along with their ID number) follows below.

**Table 3:** Thematic framework

Code	Theme	#	Categories
A	Performing the choice tasks	1	Hypothetical context
		2	Interpretation of (missing) information
		3	Experienced burden
B	Perceptions about the patients	4	Characteristics of patients
		5	Coping mechanism of participants
C	Perceptions about the condition	6	Connotations of the conditions
		7	Severity of condition
		8	Type of condition
D	Perceptions about the treatment options	9	Health gains
		10	Suitability of surgical treatment
		11	Suitability of alternative treatment
E	Stating a preference	12	Interpretation and weighting of attributes and levels
		13	Perceived consequences of choices

### Theme A: Performing the choice tasks

This theme comprises three categories describing the hypothetical context of the choice tasks, participants’ interpretation of (missing) information provided in the choice tasks and the experienced burden of completing the tasks.

While performing the choice tasks, participants commented on the hypothetical context in which they were asked to prioritise patients for scarce surgical care. For example, one participant mentioned that although it was stated that the patient suffering from breast cancer would not die within the first 5 years after surgery, they *“could imagine that this would be different in real life”* (ID3). Some participants also remarked on the information presented to them on screen. They expressed they lacked sufficient knowledge to make an informed choice between the patients, which made stating a preference challenging. One participant mentioned that they *“really struggle[d] to make these kinds of decisions from an ethical perspective because [they didn’t] have the full picture”* (ID4). One participant mentioned the importance of information on how long the patients had to wait after the first patient was prioritised and operated on: *"What also plays a role, and what is not stated here, is how much longer it will take. If it’s the next day, that’s a completely different story than if it’s in 3 months…"* (ID4). Some other participants asked for additional information on a range of issues pertaining to the patients (eg, their occupation, family situation and socio-economic status), their condition (eg, the level of pain associated with knee arthrosis and the stage of the tumour) and the treatment options available to patients (eg, the impact of the alternative treatment option on patients’ HRQoL).

Besides the articulated need for more information, the information that was presented during the tasks was subjected to scrutiny. Participants questioned the level of realism of information. For example, when participants considered the fact that patients do not live longer and do not die sooner due to surgery, some tended to deliberately ignore this information because it did not align with their perceptions of the condition: *“So then you can say, yes, surgery: you won't die from it or you won't live longer, but that seems a bit of a pipe dream – utopian – with breast cancer”* (ID18). Instances were also observed where participants not deliberately but accidentally forgot or ignored information presented to them in order to correctly carry out said task, such as overlooking attributes or forgetting the alternatives for surgical treatment.

The burden of completing tasks differed across participants: some found it (morally) difficult, referring to ‘playing God’, ‘playing judge’ or ‘Russian roulette’, while others found it easier to do because they knew it was not for real. The repetitiveness of tasks seemed to reduce the burden as well. When participants touched on the subject of difficulty of task completion, choosing between alternatives with the same condition was found easier to make in all cases because participants did not have to discount the (severity of the) condition in their preferences. One participant mentioned that *“given that you are dealing with the same type of people here, it is just much easier to make a choice than when you have different people in front of you. With that comes many more elements which is much harder to put into certain frames than when you are dealing with the same type of people” (ID14*). In performing the tasks, evolving insights of participants led them to reconsider their initial decision heuristics: *“Now yes, I do find it a little trickier, also because I just noticed that I was also a little conflicted about what I chose first and then again afterwards”* (ID3). In some instances, this implied that previously participants would be led by unsettling emotions in making their preferences; they switched to a more rational approach of stating their preferences. Some participants expressed frustration about the role of physicians, noting that patients are often made to wait, dismissed or *“have to beg”* for treatment (ID5). At the same time, participants recognised that physicians cannot predict the future—they *“don’t have a crystal ball”* (ID5). Transparency was considered crucial: participants valued clear explanations for delays, honesty about prioritisation decisions and keeping promises (eg, when someone is told they will be treated) (ID1). These views were often grounded in participants’ personal experiences. Participants acknowledged the difficulty of prioritisation decisions for physicians. While they could understand the emotional desire to prioritise certain patients, they also believed that physicians should not allow emotions to influence such decisions.

### Theme B: Perceptions about the patients

This theme comprises the characteristics of patients and the coping mechanisms of participants.

Factors that could have influenced the preferences are related to either personal circumstances or experiences that factor into the participants’ preferences. These experiences, and participants’ representations of patients, influence how they judge the several scenarios presented to them. Personal considerations, such as the one below, were discounted in the decision by some, whereas others stated that these should not be allowed to influence their choice: *“I have two children myself. And as a parent you want them to be helped as soon as possible, but yes, those children also have a grandmother and if that grandmother has breast cancer then you want her to be helped as soon as possible”* (ID4). Participants additionally pondered the social background of patients; habits, behaviours and their consequences (eg, smoking, physical exercise, obesity); fitness level; self-sufficiency; complications of surgical treatment, probability of success of treatment and contribution to society (work participation) as factors and to what degree patients suffered. Saliently, a dividing line emerged between patients who have reached retirement age and those that have not, which was associated with the need for prioritisation due to the latter’s participation in and contribution to society: *“Yes, so yes, I think at 60 you're still very much in life and 80 will no doubt be at least less so. A lot less, I think”* (ID17). However, participants differed regarding the degree to which all these considerations should be taken into account when prioritising patients.

### Theme C: Perceptions about the condition

This theme comprises three categories describing the connotations of the conditions, the severity of the condition and the type of condition.

The perception of the condition was shaped by considering the condition in relation to the different attributes, as well as factors that were not included as attributes. For instance, the severity of breast cancer was an important emotional trigger for participants. Besides that, participants associated several characteristics with the conditions. Breast cancer was associated with the chance of metastases and recidivism, unpredictability, being life-threatening and its implications for the mental and physical condition of the patient and their immediate environment. One participant mentioned that *“for someone with cancer, yes every day is one day too many and they are really waiting for it [treatment]. It can also have a lot of impact on other things, like mental state, and on the family. So in that sense those are important factors that I take into consideration”* (ID14).

Furthermore, breast cancer was perceived as needing more acute care as opposed to deafness and knee arthrosis, which were qualified as chronic conditions. Surgery for cancer is more urgent than for deafness or arthrosis; hence, in general, long waiting lists for breast cancer are less acceptable than for the other conditions. Knee arthrosis was mainly associated with pain or inconvenience, the latter being considered normal at an elderly age by one, while the other shared negative experiences of people in their environment who suffered from arthrosis or similar conditions. Some participants trivialised knee arthrosis, while others reflected on the lack of mobility and impediment to freedom as a consequence of knee arthrosis: *“But I think that if someone is not mobile, then physically, but also mentally you just don't have freedom”* (ID21).

Perceptions on deafness were mixed, with some participants indicating that deafness during infancy would not pose significant developmental barriers to the patient, whereas others stated that infancy is a crucial phase for language development and interaction with other children. Another argument for not choosing to prioritise this condition was the fact that the infant would be used to deafness: *“And also because yes, maybe it sounds weird, but patient 1 who’s not used to anything else, I guess, then to be deaf”* (ID2).

### Theme D: Perceptions about the treatment options

This theme comprises three categories that describe health gains, suitability of surgical treatment and suitability of alternative treatment options.

In disclosing the perception of treatment options, participants talked about the suitability of surgical treatment, alternative treatment and the health gains that corresponded with treatment. Participants considered patients’ HRQoL before and after surgery, which was considered by some to be relatively high. This was considered, as well as the incremental gains due to surgery, which some participants considered to be small. One participant mentioned that patients have to be mentally healthy in order to have the strength and capacity to benefit from surgery in terms of HRQoL increase (ID5). Participants furthermore considered the life expectancy of patients, particularly in relation to breast cancer. One participant mentioned that while the condition was not life-threatening, cancer itself was still a deadly condition (ID5). Some mentioned that the choice tasks would have been easier had patients’ life expectancy been influenced by the surgery, as this would have been decisive in their choice (ID12).

Participants shared their opinions and associations with the primary treatment options for the conditions. A salient topic was the risk of complications after surgical treatment, which included long recovery times, the complications of narcosis, or early death. Participants also shared that as the age of patients increases, the risk of complications increases, diminishing the appropriateness and necessity of primary treatment at advanced age. As such, surgical treatment for knee arthrosis was considered risky: *“Maybe the patient doesn't die because of the surgery but what you often see is that because of post-surgery recovery, patients do end up dying sooner, especially after such a major surgery like knee or hip surgery, than if they had not undergone it”* (ID4).

In relation to breast cancer, the surgical treatment and its chance of success were dependent on how fast the treatment was given. Hence, this indicates the urgency of primary treatment and disqualifies the alternative treatment option as not sufficient: *“Breast cancer, 50 years old; yes, a tumour has to be removed as soon as possible otherwise it will get bigger and radiotherapy has to start as soon as possible”* (ID4).

In disclosing their thoughts about the alternative treatment options, participants considered attributes vis-à-vis the condition. For example, in breast cancer, longer waiting times and higher age were linked with increased chances of metastases. These aspects factored into the participants’ perceptions of the alternative treatment for breast cancer (radiotherapy), which was considered by many as inadequate or at least as profound as primary (surgical) treatment: *“The alternative of radiotherapy is extremely drastic on your quality of life as well, so I think that’s almost as bad as surgery”* (ID8).

The appropriateness of the alternative, physiotherapy with pain medication, was considered in relation to the patients’ age: the added value of knee replacement at advanced age was debated, and the alternative of using pain medication and doing exercises was considered appropriate. The alternative for deafness—the external hearing aid—was considered by some participants as just as appropriate as primary treatment. It was also considered by some as the best of the alternatives, compared with radiotherapy and physiotherapy. Others remarked that the external hearing aid would be clearly visible to others, which may trigger bullying by peers—certainly at young age—which would have detrimental long-term effects for the patient. Age was an important aspect for participants to consider the alternative for the cochlear implant as adequate. At a younger age, the alternative was deemed less acceptable as opposed to when patients are older. By others, it was deemed adequate because the hearing aid enabled the infant to at least hear and was a suitable bridge to a permanent solution, while waiting too long would impede the development of the infant.

### Theme E: Stating a preference

This theme comprised two categories that describe participants’ interpretation and weighting of the attributes and levels and the perceived consequences of their choices.

The interpretation of the attributes’ age, HRQoL before and after surgery and waiting time varied between participants. Age was linked to the individual’s circumstances and the extent to which they participated in society or cared for others. Additionally, advanced age—what is advanced is judged differently by participants—was associated with lower life expectancy, lower likelihood of success of surgical treatment and risk of surgical complications. Participants generally considered patients aged 70 and 80 as relatively old, which often led them to prioritise younger patients. In contrast, patients aged 50 or 60 were seen as having *“a whole life ahead of them”* (ID7).

Participants reflected on HRQoL before and after surgery, and some simplified the different levels by interpreting them as being high or low. HRQoL was found to be a subjective measure, and some participants mentioned that patients could indicate a lower HRQoL to obtain a better position on the waiting list. Moreover, assessing the HRQoL of young children (ie, in the case of deafness) was considered difficult (ID2). Therefore, it was considered an indicator that was difficult to interpret and weigh, as compared with age and waiting time. This remained the case even when participants were told that HRQoL was not based on patients’ own assessment but that of a physician. Despite participants having been informed that HRQoL was determined by the degree to which patients experience pain, discomfort, anxiety or feelings of dismalness, in their own interpretations the scope and dimensions of HRQoL deviated: “*Added to that are social and spiritual aspects that I don't think you can express in points. So that’s what makes it so difficult. Look, someone might not improve a lot physically, but knowing that the person has been helped in 6 months might already give more points to quality of life”* (ID12). The participants clearly indicated that they were aware of the potentially negative consequences their preferences had. While pondering the consequences of their preference, participants mainly related to the waiting time of the patients that were not prioritised and the consequences it could have for HRQoL or, in the case of the infant, development. Consequences of waiting were considered less severe for some patients.

Prioritising patients based on waiting time was perceived as fair by several participants. Some considered waiting time a decisive factor, while others hardly took it into account. Three months of waiting was generally seen as acceptable, but a mental boundary was described—after which waiting was no longer tolerable. Waiting time was also considered in relation to patients’ age and life expectancy. For example, waiting several months was perceived as worse for someone who is 80 years old compared with a younger person. Similarly, the same waiting period was perceived differently depending on whether a patient had a family to care for.

Although participants knew how long patients had already been waiting, they did not know how much longer they would have to wait if not prioritised. In general, the longer a patient had waited, the more likely they were to be prioritised. However, some participants argued that if a patient had already been waiting 18 months, waiting a little longer would not be problematic, as this indicated there was no urgency. One participant even suggested that such patients could be treated abroad.

Participants also discussed how the second patient in the queue should be treated. It was assumed that it would be a matter of hours or days before the second patient’s would be treated. It was also mentioned that this person should not have to go through the entire prioritisation process again unless acute cases arise. Another participant reasoned as follows: “*Yes, I choose patient 1 anyway, because then there will be room for the other one when that is done. I think, simply”* (ID5). Alternatives were weighed against each other on several dimensions: consequences for another patient if they were to receive alternative treatment, resulting in a negative choice. For example, alternative treatments for knee arthrosis or deafness were considered adequate, resulting in a preference for surgical treatment for breast cancer: “*The knee osteoarthritis can always come later because you can use pain relief and exercises first. And deafness, oh yes, the surgery, but with deafness a lot of people can manage very well with sign language anyway and so then I would still choose the surgery, to remove the tumor”* (ID5).

Choosing between patients with the same condition was generally seen as less complex. Without needing to weigh the relative severity of different conditions, participants found it easier to base their decisions on other attributes. One participant explained that when comparing *“the same type of people,”* making a choice *“felt much easier”* than when comparing individuals with different conditions, which introduced *“many more elements”* that were *“much harder to put into certain frames”* (ID14). Some participants described how their approach to the tasks evolved over time. As they moved through the series of decisions, they began to reflect on and sometimes revise their initial reasoning. One participant admitted to feeling conflicted between their early and later choices, noting, *“I just noticed that I was a little conflicted about what I chose first and then again afterwards”* (ID3). In such cases, participants described a shift away from emotionally driven responses toward more deliberate and rational reasoning. Costs of surgery and alternative treatments were also considered in stating the preference. “*Now I also have to be honest: […] irradiating a tumour is also quite pricey I'm thinking”* (ID19). There were also references to medical consumerism, as in the notion of simply *“taking a new knee”* (ID5).

## Discussion

This study aimed to understand the considerations behind preferences of citizens in the context of prioritising patients. Five themes were identified that systematically examined these considerations. Our study shows that when completing DCE-based choice tasks, participants relied not only on the presented attributes but also integrated a wide array of contextual, emotional and ethical factors into their decision-making, which is in line with previous research.^2^ Participants drew from a combination of heuristics, factoring in severity of the condition, age, quality of life, waiting time or an ingenious blend of these elements, making individual preferences very context-dependent. Our findings further show that participants found the choice tasks challenging and often preferred, and relied on, personal experience instead of information provided to them in the choice tasks. Ethical concerns and emotional responses influenced choices, though comparisons were easier when patients had similar conditions.

This study additionally served to refine a DCE administered among 1046 members of the Dutch public.^22^ Our findings indicated that participants tended to prioritise patients with breast cancer over those with knee arthrosis or deafness, and in some cases, this preference was consistent across all choice tasks. Our study may provide some explanation for why breast cancer was prioritised over the two other conditions. First, the profound impact of breast cancer on everyday life, previous experiences with breast cancer and its progressive nature all contributed to its perceived urgency. Breast cancer was associated with the chance of metastases and recidivism, making its disease progression highly unpredictable. Furthermore, its implications for the mental and physical condition of the patient and their immediate environment were considered, which might be explained by previous experiences participants had. Second, attributes were weighted differently for breast cancer. For instance, waiting for breast cancer surgery was considered more impactful than waiting for knee arthrosis surgery. Previous research shows that fear of cancer could influence priority preferences of participants.^24^ Our findings corroborate this assumption, showing that cancer evokes strong emotions in the participants, steering them towards a preference for prioritising this condition.

Although informing the design of a subsequent study was not the primary aim of the current study, the findings yielded methodological insights that were used to design a subsequent DCE study.^22^ To further investigate priority preferences across conditions, we developed a design consisting of choice tasks in which participants were asked to prioritise one of three patients with breast cancer, deafness or knee osteoarthritis. The design included alternatives with overlapping condition labels within the same choice task (eg, patient 1: breast cancer, patient 2: breast cancer, patient 3: deafness). As a result, even participants with a consistent preference for prioritising patients with breast cancer were required to make trade-offs between the attributes and levels describing the patients, rather than relying solely on condition labels when making their choices. Another finding was that participants were less likely to prioritise older patients, except for deafness.^22^ Our study offers some explanations. First, participants associated advanced age—which varied in definition among participants—with lower life expectancy, reduced surgical success and higher complication and recovery risks. Second, a key distinction arose between retirees and non-retirees, with the latter more often prioritised because of their presumed societal participation.

An unexpected finding was that deaf patients are least likely to be prioritised, especially at younger ages.^22^ We observed that participants see hearing aids as a viable alternative to the primary treatment (ie, cochlear implant). However, at a younger age the hearing aid was perceived as a less viable alternative. Paradoxically, deafness at a younger age is less likely to be prioritised as evidenced by the DCE, while the alternative is deemed less viable. Previous research demonstrated a significant preference of participants to allocate scarce resources to children.^13^ However, based on our findings, this does not necessarily mean that young patients that experience deafness will receive primary treatment, because the alternative treatment option was considered by some as just as adequate.

The attributes included warrant some reflection. Many participants indicated that they found the HRQoL levels acceptable, which may have to do with the fact that participants on average gave themselves HRQoL scores on the VAS that are within the range of HRQoL scores in the DCE. Further, little is known about prioritisation based on waiting time. Although participants considered it important to help those who had been on the waiting list longer, this preference was weighed against the severity of the condition and other attributes. As a result, the first-come-first-served principle was often set aside by participants in favour of considerations of age, life expectancy and health gains. In addition, time on the waiting list was also considered in relation to the patient’s remaining life expectancy and age.

Although not directly meeting the aim of the study, we considered it striking that some participants tried to relate to the physician’s position in stating a preference. On the one end, this meant that participants identified and empathised with the physician in having to make ‘an impossible choice’, whereas others had less confidence in leaving these choices to physicians because they would supposedly prioritise their ‘own’ patient. This did not seem to directly influence the preferences but may inform the resistance to the choices physicians make. Further research is needed to map the perspective of citizens on priority-setting by physicians in times of acute and unanticipated scarcity.

The findings of this study demonstrate the value of combining think-aloud methods with DCE-based choice tasks to better understand how participants interpret attributes, make trade-offs and reason through prioritisation decisions. Whereas quantitative DCEs primarily estimate preference structures, think-aloud methods can provide complementary insight into the contextual, emotional, ethical and broader societal considerations underlying participants’ choices, highlighting that their choices reflected more than trade-offs between the attributes presented in the choice tasks alone. Such insights may help refine DCE design, improve interpretation of DCE findings and identify considerations that may not be fully captured through predefined attributes alone.

### Limitations and future research

This study is not without limitations. Our sample showed a slight overrepresentation of people with tertiary education and underrepresentation of people aged 65–80 compared with the entire Dutch population. The sample furthermore did not include people with lower education levels. These limitations may affect the generalisability of the findings, as education and age can influence views on healthcare prioritisation. However, we have diversified our sample through making use of profiles that account for people’s lifestyles and ideological preferences. In this way, we hope to have captured a broad range of considerations given our small sample size. The fact that a third of the participants consistently choose breast cancer could mean that future studies should consider involving a different condition than breast cancer in such DCEs. This indeed confirmed that people seem to state preferences based on the lethality of the condition, thus risking overlooking other attributes that play a role in the DCE. However, most participants expressed preferences that were informed by a more complex consideration of attributes. Our findings further indicate that when participants *do* prioritise based on a utilitarian mental framework, they maximise the health gains of the individual, not the population. On this point, the framework used by participants differs from the framework in the proposed model to support prioritising surgical capacity that has the goal of maximising population health as opposed to individual health. This may have been a shortcoming of this study which could be addressed in future research. Finally, this study was qualitative in nature, and the sample size was intended to support in-depth exploration of participants’ reasoning processes rather than to quantitatively estimate population preferences for prioritising patients in the face of scarce surgical capacity in the Netherlands. Future research could build on these findings by applying the think-aloud approach to examine how citizens reason about the prioritisation of emerging healthcare technologies and other complex healthcare allocation decisions, thereby further informing the design and interpretation of quantitative preference studies.

### Recommendations for policy

Determining which patients receive priority may not be the sole responsibility of healthcare professionals but also falls within the purview of policymakers. To enhance the legitimacy and acceptability of priority-setting policy, clinical decision support models could be informed by the preferences and perspectives of citizens. In order to strike an appropriate balance between various moral and ethical principles, it may be important to consider multiple perspectives to prevent simplifying context-specific issues into generalised principles or guidelines.^25^ Though previous research indicates that in times of scarcity, patient representatives^5^ may prefer a utilitarian approach to prioritisation of patients, its practical operationalisation may have a different outcome. Our findings suggest that citizens—as opposed to patient representatives and professionals—also draw from principles of prioritarianism.

## Supplementary material

10.1136/bmjopen-2025-115112 online supplemental file 1

10.1136/bmjopen-2025-115112 online supplemental file 2

10.1136/bmjopen-2025-115112 online supplemental file 3

## Data Availability

No data are available.
