# Supplementary material for: Understanding citizens’ preferences for prioritising patients in the face of scarce surgical capacity in the Netherlands: a think-aloud study
Source: BMJ Open. 2026 Jul 7;16(7):e115112. doi: 10.1136/bmjopen-2025-115112 (PMC13343013; doi:10.1136/bmjopen-2025-115112)
Supplement: online supplemental file 1 [file bmjopen-16-7-s001.pdf]

Supplementary Material S1

Table S1 Overview of conditions, surgical procedures, and alternative treatments

	Condition	Surgical procedure	Alternative treatment
1	Benign prostatic hyperplasia	Transurethral resection of the prostate	Conservative
2	Breast cancer, T1–2	Lumpectomy	Radiotherapy
3	Deafness (paediatric population)	Cochlear implantation	External hearing aid
4	Hip osteoarthritis	Hip replacement	Pain relief and exercises
5	Knee osteoarthritis	Knee replacement	Pain relief and exercises
6	Laryngeal cancer T1–2	Surgical resection	Radiation therapy
7	Locally advanced prostate cancer	Prostatectomy	Hormone therapy
8	Multivessel disease	Coronary artery bypass graft	Optimal medical therapy
9	Persistent atrial fibrillation	Maze procedure	Cardiac ablation
10	Symptomatic bradycardia	Pacemaker implantation	Optimal medical therapy
11	Ventricular arrhythmias	Implantable cardioverter–defibrillator	Optimal medical therapy

Supplementary Material S2

Table S2. Description of Mentality™ profiles

Profile	Description	n (%)
Cosmopolitans	Critical world citizens who integrate postmodern values of development and experience with modern values of success, materialism and enjoyment.	4 (16.0)
Postmaterialists	Socially critical idealists who want to develop themselves, take a stand against social injustice, and stand up for the environment.	3 (12.0)
New conservatives	Liberal-conservative upper class that embraces technological development but is reluctant to social and cultural innovation.	3 (12.0)
Traditionals	Moralistic, dutiful, and status quo-oriented bourgeoisie that clings to traditions and material possessions.	2 (8.0)
Modern mainstream	Conformist and status-sensitive bourgeoisie that aims for a balance between tradition and modern values such as consumption and enjoyment.	4 (16.0)
Impulsive individualists	The spontaneous consumer who primarily seeks a pleasurable and comfortable life.	3 (12.0)
Social climbers	Career-oriented individualists with an outspoken fascination for social status, new technology, risk, and excitement.	3 (12.0)
Postmodern hedonists	Pioneers of the experience culture, in which experimentation and breaking with moral and social conventions have become goals in themselves.	3 (12.0)

Source: https://www.motivaction.nl/en/mentality.

Supplementary Material S3

Interview protocol

1. Opening

1. Welcome
2. Offer beverage
3. Explain interview objective and procedure
4. Signing of informed consent
5. Answer any questions of participant

2. Think-aloud protocol

Thank you for participating in this study. This interview will last approximately 60 minutes. During the interview, we ask you to complete two sets of 12 choice tasks while you think aloud, meaning that you are asked to verbalize any thought that you have during the completion of the tasks.

During the interview, we will ask you to choose between different patients, with the same or different conditions. These include babies who are deaf, adults/women with breast cancer and elderly people with knee osteoarthritis. Suppose there is only one spot available in the operating room, you will choose (for the doctor) which of the three patients will be operated on immediately.

Since we would like to know why you make a particular choice, we would like to ask you to think out loud. This is important, because your ideas about this will be used to create a model that will help doctors and policymakers make good choices when there is not enough room in the hospital for everyone, for example in a major disaster or epidemic.

We would like to ask you to share all your thoughts with us – we will treat your data confidentially [informed consent form to be read]. While completing the questionnaire, I may ask you to continue to speak your thoughts [see Think-aloud prompts]. If there are thoughts you would rather not share with us because you find it unpleasant, please do not share them.

In between tasks and afterwards, I will ask some questions.

Think-aloud prompts

Participant has not spoken thoughts for 10 seconds: "Please continue to speak your thoughts aloud."

Participant says few or considers health states silently: "Can you please say out loud what you were just thinking about?"

Participant speaks unclearly: "Can you please repeat what you just said?"

3. Answer any questions of the participant

4. Practicing think-aloud through 'count the windows' exercise

Suppose you are in your house, walking through your house and counting the windows. Can you do this? Describe the windows, what do they look like? Can they open, close? What is the view? How big is the window? What is the next window?

5. Start recording device

DCE tasks

1. Answer the participant's questions about the tasks.

2. Remind the participant of the think-aloud protocol

3. Complete the short questionnaire

4. Practice DCE tasks

5. Two sets of DCE tasks

6. Using think-aloud prompts during DCE tasks

Follow-up questions after first set of tasks

Now that you have completed the first 12 choice tasks, I have a few more questions for you.

1. What was it like for you to make these choices out loud?

2. Is there anything that you did not say out loud, but that you feel is important to still mention?

3. What was the most important difference for you between the three patients? Can you explain how that affected your choices?

4. How did you imagine the patients when you made the tasks? Can you explain how this influenced your choice? Would your choice have been different if you or one of your acquaintances had been one of the patients?

5. Were there any other factors that influenced your choices?

6. Are there other factors that should be considered by physicians in their choice?

Closing questions after second set of tasks

Now that you have completed the last 12 choice tasks, I have a few closing questions for you.

1. Can you name other reasons/things we did not put in the descriptions that you think are important when it comes to priority in operations? [To identify which attributes we missed].

2. What could be possible consequences of your choices? [If A is chosen, then B and C will not be helped immediately].

3. What else do you think is important when it comes to choosing between these patients? [Again to identify what attributes we missed]

4. Is there anything else you would like to give to people who use this information to make choices in the hospital?

5. Would that mean that someone who smokes should not be helped first? Or someone who is no longer productive, would that be a reason not to put someone ahead of them?
