# Supplementary material for: Understanding citizens’ preferences for prioritising patients in the face of scarce surgical capacity in the Netherlands: a think-aloud study
Source: BMJ Open. 2026 Jul 7;16(7):e115112. doi: 10.1136/bmjopen-2025-115112 (PMC13343013; doi:10.1136/bmjopen-2025-115112)
Supplement: online supplemental file 3 [file bmjopen-16-7-s003.pdf]

Supplementary Material S5

Table S5.1 Sample characteristics (N=25)

	n (%)	Mean (SD)	Min	Max
Age (Years)		45.2 (15.2)	15	75
Sex (Female)	13 (52.0)			
Education level[b]				
Medium	12.0			
High	88.0			
Main occupation				
Student	2 (8.0)			
Volunteer	2 (8.0)			
Employed	16 (64.0)			
Retired	3 (12.0)			
Rather not say	1 (4.0)			
Children (Yes)	12 (48.0)			
ED-5D-5L index		0.92 (0.12)	0.46	1.00
EQ-5D VAS		83.4 (10.6)	55	100
Interview duration (Minutes)		52.8 (10.5)	36	81

VAS, visual analogue scale (ranging from o “dead” to 100 “full health”).

[a] Medium = middle vocational and secondary school, High = higher vocational and academic education;

[b] EQ-5D-5L index value is calculated using the EQ-5D-5L value set for the Netherlands (20).
