# Supplementary material for: Understanding citizens’ preferences for prioritising patients in the face of scarce surgical capacity in the Netherlands: a think-aloud study
Source: BMJ Open. 2026 Jul 7;16(7):e115112. doi: 10.1136/bmjopen-2025-115112 (PMC13343013; doi:10.1136/bmjopen-2025-115112)
Supplement: Supplementary file 4 [file bmjopen-2025-115112.reviewer_comments.pdf]

PEER REVIEW HISTORY

BMJ Open publishes all reviews undertaken for accepted manuscripts. Reviewers are asked to complete a checklist review form and are provided with free text boxes to elaborate on their assessment. These free text comments are reproduced below.

ARTICLE DETAILS

Title (Provisional)

Understanding citizens' preferences for prioritizing patients in the face of scarce surgical capacity in the Netherlands: A think-aloud study

Authors

Reindersma, Thomas; van Alphen, Anouk; Reckers-Droog, Vivian

VERSION 1 - REVIEW

**Reviewer**	**1**
**Name**	**Craig, Benjamin**
**Affiliation**	**University of South Florida, Economics**
**Date**	**07-Mar-2026**
**COI**	**I am a member of the EuroQol Group.**

<p>

This manuscript currently misrepresents its primary contribution. While framed as a Discrete Choice Experiment (DCE), the study lacks the necessary statistical power and sample size (N=25) to support that classification. Instead, this work represents formative qualitative research (a think-aloud study) designed to support a larger DCE. The authors acknowledge that these results informed a separate, published study; therefore, the value of this paper lies in its methodological contribution to the development of preference study protocols.</p>

<p>I recommend the authors rewrite the paper as a case study, focusing on how qualitative understanding informs DCE design and how the methodology can be improved.</p>

<hr>

<p>

</p>

<p>

The authors should rewrite the paper following the guidelines provided by

for reporting formative qualitative research (

, 13(1), 121-136). Specifically, the authors must demonstrate how the qualitative themes identified were actionable. How did these findings lead to specific changes in the decision scenario, task design, or the descriptive system (attributes and levels) of the subsequent DCE?</p>

Hollin IL, Craig BM, Coast J, Beusterien K, Vass C, DiSantostefano R, Peay H. Reporting Formative Qualitative Research to Support the Development of Quantitative Preference Study Protocols and Corresponding Survey Instruments: Guidelines for Authors and Reviewers. Patient. 2020 Feb;13(1):121-136. doi: 10.1007/s40271-019-00401-x. PMID: 31840215.

<p>

To increase the paper's contribution, I suggest conducting a formal thematic analysis of the interview transcripts to identify which interview prompts were more or less informative. This would shift the paper from a simple pilot report to a methodological contribution that identifies gaps in interview scripts and improves future formative research.</p>

<p>

The current references are too generic. The authors should cite the specific literature dedicated to formative qualitative methods and remove broader citations that do not support the target literature of this study.</p>

<p>

Following

, the choices described here more closely represent "commitments" than "preferences." For these to be categorized as preferences, the alternatives must have direct consequences within the respondent’s concern (e.g., "Imagine you have three children with different health problems"). Please adjust the conceptual framing accordingly.</p>

Sen, Amartya K. (1977). “Rational Fools: A Critique of the Behavioral Foundations of Economic Theory.” Philosophy & Public Affairs, 6(4), 317–344.

<hr>

<p>

</p>

<p>1.

A DCE is an experiment comprising a series of choice tasks, not a "task" itself. In this case, the pick-one tasks featured two or three labeled alternatives and excluded an opt-out.</p>

<p>2.

Update the abstract to follow standard reporting practices for formative qualitative research rather than quantitative experiments.</p>

<p>3.

Provide the experimental design as supplemental material and include the registration number for the EQ-5D-5L from the EuroQol Foundation.</p>

<p>4.

Table 2 belongs in an online supplement; the text description provided in the manuscript is sufficient.</p>

Reviewer 2

Name Estrada-Orozco, Kelly

Affiliation Universidad Nacional de Colombia, Health Technologies and Policies Assessment Group, School of Medicine

Date 25-Mar-2026

COI None

This manuscript explores citizens' preferences regarding the prioritisation of patients and health conditions—including surgical interventions—in contexts of constrained surgical capacity. The topic is highly relevant, particularly in the current global context where health systems face increasing pressure on resource allocation and prioritisation decisions.

The study employs a think-aloud methodology, which is both innovative and particularly well-suited to the research objective. This approach allows for a deeper understanding of how individuals reason through prioritisation decisions, capturing not only outcomes but also the cognitive processes underlying them. The application of this method is rigorous and clearly described, making it accessible even to readers who may not be familiar with qualitative or mixed-methods approaches.

One limitation relates to the representativeness of the participant sample, which may affect the generalisability of the findings. However, the authors appropriately acknowledge this limitation and provide sufficient transparency regarding the characteristics of the sample and recruitment process.

From a formal and structural perspective, the manuscript is well written, coherent, and easy to follow. The authors successfully communicate complex methodological concepts in a way that is understandable to a broad audience.

I particularly appreciated the contribution of this work in bringing forward citizens' perspectives into prioritisation processes—an area that is often dominated by clinical, economic, or utilitarian frameworks. This study adds important value by highlighting how

societal values and reasoning can inform more inclusive and legitimate decision-making in healthcare.

Overall, this is a strong and insightful manuscript that makes a meaningful contribution to the literature on health prioritisation and resource allocation.

VERSION 1 - AUTHOR RESPONSE

REVIEWER #1

GENERAL COMMENTS:

This manuscript currently misrepresents its primary contribution. While framed as a Discrete Choice Experiment (DCE), the study lacks the necessary statistical power and sample size (N=25) to support that classification. Instead, this work represents formative qualitative research (a think-aloud study) designed to support a larger DCE. The authors acknowledge that these results informed a separate, published study; therefore, the value of this paper lies in its methodological contribution to the development of preference study protocols. I recommend the authors rewrite the paper as a case study, focusing on how qualitative understanding informs DCE design and how the methodology can be improved.

Response: We thank the reviewer for this thoughtful comment and agree that the primary contribution of this manuscript lies in the qualitative exploration of citizens' reasoning and decision-making processes while completing choice tasks, rather than in estimating preference weights through a quantitative discrete choice experiment (DCE). Our intention was not to present this study as a fully powered quantitative DCE, but rather as a think-aloud study using DCE choice tasks to gain insight into the considerations underlying prioritization decisions in the context of scarce surgical capacity. In response to the reviewer's comment, we revised the manuscript to more clearly position the study as a qualitative think-aloud study that used DCE choice tasks as an elicitation tool.

Next to that, although the findings informed the refinement of a subsequent large-scale DCE study, the goal was not necessarily to inform development of the large-scale DCE study. Rather, the aim of this study was to examine citizens' preferences and the considerations they regard as important in decisions on the prioritization of patients for scarce surgical capacity. Therefore, the work does not represent formative qualitative research.

We also strengthened the discussion of the methodological contribution of combining think-aloud methods with DCE choice tasks to better understand citizens' reasoning processes and to inform preference study design.

MAJOR REVISIONS:

1. REFRAMING THE CONTRIBUTION

The authors should rewrite the paper following the guidelines provided by Hollin et al. (2020) for reporting formative qualitative research (The Patient, 13(1), 121-136). Specifically, the authors must demonstrate how the qualitative themes identified were actionable. How did these findings lead to specific changes in the decision scenario, task design, or the descriptive system (attributes and levels) of the subsequent DCE? Hollin IL, Craig BM, Coast J, Beusterien K, Vass C, DiSantostefano R, Peay H. Reporting Formative Qualitative Research to Support the Development of Quantitative Preference Study Protocols and Corresponding Survey Instruments: Guidelines for Authors and Reviewers. Patient. 2020 Feb;13(1):121-136. doi: 10.1007/s40271-019-00401-x. PMID: 31840215.

Response: We thank the reviewer for this thoughtful suggestion and for drawing our attention to the reporting guidance by Hollin et al. (2020). Following a similar request from the editor, we completed the COREQ checklist and supplemented the manuscript with several details to further improve the transparency and completeness of reporting.

As explained in our response to the reviewer's general comment, our intention was to present this study as a qualitative think-aloud study using DCE-based choice tasks to gain insight into the considerations underlying prioritization decisions in the context of scarce surgical capacity. While the findings informed the design of a subsequent large-scale DCE study, this was not the primary purpose of the study and, hence, is now presented as a secondary contribution. To address the reviewer's comment, we added a paragraph to the Discussion section describing how the findings of the current study informed the design of the subsequent DCE study. Specifically, the think-aloud interviews revealed that some participants relied heavily on condition labels when making prioritization decisions. This insight informed the design of the subsequent DCE, in which alternatives with overlapping condition labels were included within the same choice task to encourage respondents to make trade-offs between patient characteristics rather than relying solely on condition labels.

2. METHODOLOGICAL RIGOR AND THEMATIC ANALYSIS

To increase the paper's contribution, I suggest conducting a formal thematic analysis of the interview transcripts to identify which interview prompts were more or less informative. This would shift the paper from a simple pilot report to a methodological contribution that identifies gaps in interview scripts and improves future formative research.

Response: We thank the reviewer for this thoughtful suggestion. The primary aim of this study was to explore how participants reasoned about prioritization decisions while completing DCE-based choice tasks, rather than to evaluate the informativeness of specific

interview prompts or the interview guide itself. We therefore did not conduct a separate thematic analysis focused on individual prompts.

Nevertheless, we agree that the study offers methodological insight into the use of think-aloud methods in the context of DCE development and interpretation. To further emphasize this contribution, we strengthened the Discussion section by elaborating on how think-aloud methods can help identify contextual, emotional, and ethical considerations that may not be fully captured through predefined DCE attributes alone.

3. LITERATURE AND CITATIONS

The current references are too generic. The authors should cite the specific literature dedicated to formative qualitative methods and remove broader citations that do not support the target literature of this study.

Response: We thank the reviewer for this comment. We agree that, given the qualitative nature of this study, it is important to clearly position the study within the think-aloud and qualitative methodology literature. The manuscript already included several references on think-aloud methods and qualitative approaches in the context of DCEs and health state valuation. To further strengthen the methodological framing of the study, we added additional references specifically focused on think-aloud methodology (i.e., Van Someren et al., 1994; Willis, 2005) in the Introduction and Methods sections.

4. CONCEPTUAL FRAMEWORK (PREFERENCES VS. COMMITMENTS)

Following Sen (1977), the choices described here more closely represent "commitments" than "preferences." For these to be categorized as preferences, the alternatives must have direct consequences within the respondent's concern (e.g., "Imagine you have three children with different health problems"). Please adjust the conceptual framing accordingly. Sen, Amartya K. (1977). "Rational Fools: A Critique of the Behavioral Foundations of Economic Theory." Philosophy & Public Affairs, 6(4), 317–344.

Response: We thank the reviewer for this thoughtful comment and for referring us to the work of Sen. We agree that prioritisation decisions regarding scarce healthcare resources may reflect broader moral, ethical, and societal considerations in addition to individual self-interest. Indeed, one of the key findings of our study is that participants incorporated contextual, emotional, and ethical considerations into their decision-making.

At the same time, we use the term "preferences" in line with established terminology in the health economics and stated preference literature on healthcare prioritisation and resource allocation, where the term is commonly used to refer to choices regarding the allocation of

scarce healthcare resources. We therefore retained this terminology in the manuscript, while further clarifying in the Discussion that participants’ choices reflected broader moral and ethical considerations alongside trade-offs between attributes presented in the choice tasks.

MINOR POINTS:

1. TERMINOLOGY: A DCE is an experiment comprising a series of choice tasks, not a "task" itself. In this case, the pick-one tasks featured two or three labeled alternatives and excluded an opt-out.

Response: We appreciate the reviewer’s comment and revised the terminology accordingly by replacing “DCE task” with “choice task” throughout the manuscript. We also clarified in the Abstract and Methods section that the individual choice tasks did not include an opt-out option.

2. ABSTRACT: Update the abstract to follow standard reporting practices for formative qualitative research rather than quantitative experiments.

Response: We thank the reviewer for this comment. In response, we revised the Abstract to more clearly reflect the qualitative and exploratory nature of the study. Specifically, we clarified that this was a think-aloud study using DCE-based choice tasks to explore how participants reasoned about prioritization decisions, rather than a quantitative DCE study aimed at estimating preference weights.

3. DATA TRANSPARENCY: Provide the experimental design as supplemental material and include the registration number for the EQ-5D-5L from the EuroQol Foundation.

Response: As requested, we now provide the experimental design as Supplementary Material S4 and the EuroQol Research Foundation registration number for the EQ-5D-5L in the Methods section.

4. FORMATTING: Table 2 belongs in an online supplement; the text description provided in the manuscript is sufficient.

Response: Following the reviewer’s comment, we moved (the revised version of) Table 2 from the manuscript to Supplementary Material S5.

REVIEWER #2

This manuscript explores citizens' preferences regarding the prioritisation of patients and health conditions—including surgical interventions—in contexts of constrained surgical capacity. The topic is highly relevant, particularly in the current global context where health systems face increasing pressure on resource allocation and prioritisation decisions. The study employs a think-aloud methodology, which is both innovative and particularly well-suited to the research objective. This approach allows for a deeper understanding of how individuals reason through prioritisation decisions, capturing not only outcomes but also the cognitive processes underlying them. The application of this method is rigorous and clearly described, making it accessible even to readers who may not be familiar with qualitative or mixed-methods approaches.

Response: We thank the reviewer for these positive comments and for recognizing the relevance of the topic and the suitability of the think-aloud methodology for addressing the study objective. We also appreciate the reviewer's recognition of the rigor and clarity with which the methodology was described.

One limitation relates to the representativeness of the participant sample, which may affect the generalisability of the findings. However, the authors appropriately acknowledge this limitation and provide sufficient transparency regarding the characteristics of the sample and recruitment process.

Response: We thank the reviewer for this comment. We are pleased that the reviewer considers the limitations regarding the representativeness of the sample to have been appropriately acknowledged and that sufficient transparency was provided regarding the participant characteristics and recruitment process.

From a formal and structural perspective, the manuscript is well written, coherent, and easy to follow. The authors successfully communicate complex methodological concepts in a way that is understandable to a broad audience.

Response: We appreciate the reviewer's positive assessment of the clarity, structure, and accessibility of the manuscript. We are pleased that the methodological approach and findings were considered understandable and coherent for a broad audience.

I particularly appreciated the contribution of this work in bringing forward citizens' perspectives into prioritisation processes—an area that is often dominated by clinical, economic, or utilitarian frameworks. This study adds important value by highlighting how societal values and reasoning can inform more inclusive and legitimate decision-making in healthcare.

Response: We thank the reviewer for highlighting this contribution of our study. One of the aims of this work was indeed to provide insight into how citizens reason about prioritisation decisions, thereby complementing more clinically or economically oriented approaches to healthcare decision-making.

VERSION 2 - REVIEW

**Reviewer**	**1**
**Name**	**Craig, Benjamin**
**Affiliation**	**University of South Florida, Economics**
**Date**	**26-Jun-2026**
**COI**	

A qualitative think-aloud study can gain insight into the considerations underlying prioritization decisions in the context of scarce surgical capacity. However, it is more common that a quantitative study be designed and conducted for this purpose. Please clarify this perspective in the introduction and through out.

1. More specifically, I ask that the author's clarify when a quantitative, mixed, and qualitative study is warranted for this purpose. As they state, this study was not conducted to be formative. If not, why did they choose qualitative? Is qualitative evidence alone sufficient to measure preferences? They may compare their methods with those of MCDA and other small-sample methods for measuring preferences. For example, a few sentences saying that an initial qualitative study may be appropriate within any topic; it may be dually informative and aid in future designs (i.e., formative).

I very much appreciate the integration of think-aloud methods with DCE, but I do not understand the purpose here if it is not formative. It just seems like you skipped a step. If this study is not formative, where is the formative study to inform this one? I almost want to call it a qualitative pilot study, but that would again imply formative. Overall, I am still confused and ask that you clarify.

Lastly, if formative qualitative research was a secondary aim, the paper should still follow the report guidance and include how it helped, correct? Maybe an appendix?

2. Thank you. You might highlight this further as future work, particularly with reference to emerging technologies.

3. Thank you.

4. As for Sen, there is a lost opportunity to just say "preference" and wave hands. Due to its qualitative nature, this paper would be enhanced by a more nuanced introduction and interpretation (within the context of economics).

Furthermore, I reiterate my request that the paper present a more robust conceptual framework in the introduction (not discussion) that clarifies the linkages mentioned in the review response. This will also help the reader better understand the objective of this study (i.e., what is the respondent's role? self-interest only? altruism? commitments?) and what guided its design (e.g., sample selection). I do not know you well enough to infer your theory section from your past work (unlike Sen).

5. I believe that the minor points have been addressed.

VERSION 2 - AUTHOR RESPONSE

REVIEWER #1

1. A qualitative think-aloud study can gain insight into the considerations underlying prioritization decisions in the context of scarce surgical capacity. However, it is more common that a quantitative study be designed and conducted for this purpose. Please clarify this perspective in the introduction and throughout. More specifically, I ask that the author's clarify when a quantitative, mixed, and qualitative study is warranted for this purpose. As they state, this study was not conducted to be formative. If not, why did they choose qualitative? Is qualitative evidence alone sufficient to measure preferences? They may compare their methods with those of MCDA and other small-sample methods for measuring preferences. For example, a few sentences saying that an initial qualitative study may be appropriate within any topic; it may be dually informative and aid in future designs (i.e., formative).

Response: We thank the reviewer for this comment. We agree that quantitative preference elicitation methods, such as discrete choice experiments (DCEs), are often used to estimate preference weights and the relative importance of attributes. However, the primary aim of the current study was not to estimate preference weights, but to gain an in-depth understanding of how citizens reason about prioritization decisions and which considerations they regard as important in this context. A qualitative think-aloud approach was therefore considered the most appropriate methodology, as it enables exploration of participants' reasoning processes, interpretation of attributes, and the contextual, emotional, and ethical considerations underlying their choices. To clarify this distinction, we added a sentence to the Introduction explaining why a qualitative think-aloud approach was chosen to address the study objective and how it complements, rather than replaces, quantitative preference elicitation methods such as DCEs.

I very much appreciate the integration of think-aloud methods with DCE, but I do not understand the purpose here if it is not formative. It just seems like you skipped a step. If this study is not formative, where is the formative study to inform this one? I almost want to

call it a qualitative pilot study, but that would again imply formative. Overall, I am still confused and ask that you clarify.

Response: We thank the reviewer for this thoughtful comment and appreciate the opportunity to clarify the purpose of the study. We agree that think-aloud methods are frequently used as part of the formative development of quantitative preference studies. However, this was not the primary purpose of the current study. Rather, our primary objective was to gain an in-depth understanding of how citizens reason about prioritization decisions in the context of scarce surgical capacity, including the considerations they regard as important, how they interpret the information presented, and how they make trade-offs between competing considerations. We believe this constitutes a worthwhile research objective in its own right, irrespective of whether the findings are subsequently used to inform the development of a quantitative preference study. As mentioned in our previous response, we added a sentence to the Introduction explaining why a qualitative think-aloud approach was the most appropriate methodology for addressing the study objective and how it complements, rather than replaces, quantitative preference elicitation methods such as DCEs. We also clarified that, although the findings informed the design of a subsequent DCE study, this represents a secondary methodological contribution rather than the primary purpose of the study. We hope these revisions make the purpose of the study and its relationship to the subsequent quantitative preference study clearer for the readers of BMJ Open.

Lastly, if formative qualitative research was a secondary aim, the paper should still follow the report guidance and include how it helped, correct? Maybe an appendix?

Response: We thank the reviewer for this suggestion. We agree that, when the primary purpose of a study is to conduct and report formative qualitative research in support of a quantitative preference study, reporting guidance such as that proposed by Hollin et al. (2020) is highly valuable. However, in the current study, the primary aim was to explore how citizens reason about prioritization decisions in the context of scarce surgical capacity. While the findings also informed the design of a subsequent DCE study, this represents a secondary methodological contribution rather than the primary aim of the research. We therefore believe it is most appropriate to report the study as a qualitative think-aloud study rather than as a formative qualitative study. To further clarify this distinction, we revised the Introduction to explain why a qualitative think-aloud approach was the most appropriate methodology for addressing the study objective and clarified that informing the design of the subsequent DCE study represents a secondary methodological contribution. In addition, as requested by the editor, we have included the COREQ checklist to support transparent reporting of the qualitative aspects of the study. We believe these revisions adequately communicate both the substantive and methodological contributions of the manuscript while maintaining its primary focus.

2. Thank you. You might highlight this further as future work, particularly with reference to emerging technologies.

Response: We thank the reviewer for this helpful suggestion. We agree that the methodological insights from this study may also be relevant for future research. To reflect this, we expanded the "Limitations and future research" section by noting that future studies could build on our findings by applying the think-aloud approach to examine how citizens reason about the prioritization of emerging healthcare technologies and other complex healthcare allocation decisions. Such work may further improve our understanding of the considerations underlying prioritization decisions and inform the design and interpretation of future quantitative preference studies.

3. Thank you.

Response: The reviewer is very welcome.

4. As for Sen, there is a lost opportunity to just say "preference" and wave hands. Due to its qualitative nature, this paper would be enhanced by a more nuanced introduction and interpretation (within the context of economics).

Response: We thank the reviewer for this thoughtful comment. We appreciate the suggestion to further strengthen the conceptual framing and interpretation of the findings. While we have chosen to retain the term "preferences," consistent with terminology commonly used in the health preference and discrete choice experiment (DCE) literature, we have further clarified the Discussion by emphasizing that think-aloud methods provide insight into the broader contextual, emotional, ethical, and societal considerations underlying participants' choices, highlighting that their choices reflected more than trade-offs between the attributes presented in the choice tasks alone. Together with the clarifications made to the Introduction regarding the study objective, we believe these revisions provide a more nuanced conceptual framing while maintaining consistency with the terminology commonly used in the relevant literature.

Furthermore, I reiterate my request that the paper present a more robust conceptual framework in the introduction (not discussion) that clarifies the linkages mentioned in the review response. This will also help the reader better understand the objective of this study (i.e., what is the respondent's role? self-interest only? altruism? commitments?) and what guided its design (e.g., sample selection). I do not know you well enough to infer your theory section from your past work (unlike Sen).

Response: We thank the reviewer for this additional comment. We hope that our responses to the previous comments, together with the corresponding revisions to the Introduction and Discussion, have clarified the conceptual framing of the study, the rationale for the qualitative think-aloud approach, and the relationship between the primary aim of the study and its secondary methodological contribution. We hope these revisions address the reviewer's remaining concerns and provide readers of BMJ Open with a clearer understanding of the study objective and design.

5. I believe that the minor points have been addressed.

Response: We thank the reviewer for the positive evaluation of the revisions previously made.
